# Inferring Landscape-Scale Land-Use Impacts on Rivers Using Data from Mesocosm Experiments and Artificial Neural Networks

**DOI:** 10.1371/journal.pone.0120901

**Published:** 2015-03-16

**Authors:** Regina H. Magierowski, Steve M. Read, Steven J. B. Carter, Danielle M. Warfe, Laurie S. Cook, Edward C. Lefroy, Peter E. Davies

**Affiliations:** 1 School of Biological Sciences, University of Tasmania, Hobart, Tasmania, Australia; 2 Centre for Environment, University of Tasmania, Hobart, Tasmania, Australia; 3 Forestry Tasmania, Hobart, Tasmania, Australia; 4 Department of Forest & Ecosystem Science, University of Melbourne, Creswick, Victoria, Australia; 5 School of Mathematics and Physics, University of Tasmania, Hobart, Tasmania, Australia; 6 Tasmanian School of Business and Economics, University of Tasmania, Hobart, Tasmania, Australia; Argonne National Laboratory, UNITED STATES

## Abstract

Identifying land-use drivers of changes in river condition is complicated by spatial scale, geomorphological context, land management, and correlations among responding variables such as nutrients and sediments. Furthermore, variations in standard metrics, such as substratum composition, do not necessarily relate causally to ecological impacts. Consequently, the absence of a significant relationship between a hypothesised driver and a dependent variable does not necessarily indicate the absence of a causal relationship. We conducted a gradient survey to identify impacts of catchment-scale grazing by domestic livestock on river macroinvertebrate communities. A standard correlative approach showed that community structure was strongly related to the upstream catchment area under grazing. We then used data from a stream mesocosm experiment that independently quantified the impacts of nutrients and fine sediments on macroinvertebrate communities to train artificial neural networks (ANNs) to assess the relative influence of nutrients and fine sediments on the survey sites from their community composition. The ANNs developed to predict nutrient impacts did not find a relationship between nutrients and catchment area under grazing, suggesting that nutrients were not an important factor mediating grazing impacts on community composition, or that these ANNs had no generality or insufficient power at the landscape-scale. In contrast, ANNs trained to predict the impacts of fine sediments indicated a significant relationship between fine sediments and catchment area under grazing. Macroinvertebrate communities at sites with a high proportion of land under grazing were thus more similar to those resulting from high fine sediments in a mesocosm experiment than to those resulting from high nutrients. Our study confirms that 1) fine sediment is an important mediator of land-use impacts on river macroinvertebrate communities, 2) ANNs can successfully identify subtle effects and separate the effects of correlated variables, and 3) data from small-scale experiments can generate relationships that help explain landscape-scale patterns.

## Introduction

Natural resource management often requires a high level of evidence from applied research so that the basis for environmental decision-making can be demonstrated and defended [1, 2]. However, identifying drivers of ecological change from survey data is difficult because landscape-scale spatial surveys can only be correlative [3]. In addition, the impacts of land-use occur through multiple indirect pathways operating at different spatial and temporal scales, responses may be non-linear or historical, and the intermediate drivers of these pathways often co-vary or act antagonistically [4–6].

Increased sediment and nutrient inputs to rivers are closely associated with the impacts of agricultural land-use [6, 7]. However, not only is it difficult to derive biologically relevant indices of sediment load and nutrient exposure (timing, duration etc.) as well as scour and habitat infilling from survey data, but these factors are often confounded in field situations [6]. For example, increased nutrient inputs from agricultural land-use can interact with increased light availability (resulting from riparian vegetation removal) to stimulate algal production and increase ecosystem respiration [8], but flow reductions associated with agriculture can lead to reduced delivery of organic material and decreased ecosystem respiration [9]. Such confounding means that poor correlations between potential stressors and ecological condition do not necessarily indicate the absence of a causal relationship [1]. Patterns could be masked by variability in other environmental variables [10] or because the technique for sampling stressors is a poor surrogate measure of the processes causing ecological change. Estimates of freshwater nutrient concentrations are again a classic example: spot water measurements are a standard approach but may be a poor indicator of more biologically relevant measurements such as nutrient load [11]. Researchers therefore need to use a “weight-of-evidence” approach to identify causal pathways of impact [3, 12].

Mesocosm experiments that attempt to mimic aspects of a real world environment under controlled conditions have great potential to identify causal pathways in ecosystems by reducing the number of uncontrolled variables and allowing inferential testing of selected variables [13]. Mesocosms are capable of producing similar communities to those in terrestrial [14], marine [15] and freshwater [16] ecosystems, and can be particularly useful for modelling ecosystem processes such as food web structure [17], community assembly [18] and ecotoxicological impacts [19]. While laboratory and field mesocosm studies represented up to a third of published ecological studies in the mid-1990s, illustrating their validity as an approach to addressing ecological questions [20], they have also been criticised due to the difficulty in extrapolating findings to larger spatial and temporal scales [21]. This difficulty arises from responses being scale-dependent [22], experiments being conducted at inappropriate scales to address ecological issues [23], and the paucity of tools to readily and quantitatively extrapolate data.

The diminution in the use of mesocosm studies has coincided with an emphasis on exploring large-scale patterns and the development of landscape ecology as a major sub-discipline of ecology [24, 25]. Analyses of landscape patterns can provide great insights into the distributions of species, communities and ecological phenomena, are often cheaper and quicker to complete than experimental field studies, and can be seen as representing the outcomes of long-running natural experiments. However, the analyses must still ultimately rely on data from natural history and empirical ecology for causal interpretations [26]. This represents a long-standing challenge in ecology: finding the tools to extrapolate findings from small-scale studies in a way that can explain or infer landscape patterns. Moment approximation [27], causal criteria analysis [28] and Bayesian Belief Networks [29] all have the potential to integrate data across scales. Here we explore the use of artificial neural networks (ANNs) trained on stream mesocosm data to diagnose causal drivers of changes in ecological condition across river landscapes in northern Tasmania, Australia.

ANNs are useful when the pattern in a data set is not known or not easily described using conventional mathematics [30, 31]. They can handle multiple, interacting variables and identify subtle patterns, giving them considerable potential to address common ecological problems. ANNs are thus appropriate for investigating land-use impacts where there are multiple interacting drivers and non-linear responses. ANNs are widely used and good technical computing software packages are available to facilitate their application. However, suitable ecological applications are few and far between (but see for example [32–34]) because it is often difficult to establish the data sets needed for ANN training and validation, especially since the more complicated the pattern recognition exercise, the more data is required.

Our overall aim was to determine whether ANNs can be a useful tool to support conclusions about the relative influence of different drivers of river condition across disparate scales. This approach requires that the relationships between nutrient or fine sediment loads and macroinvertebrate communities have common mechanisms and responses at the scale of the flowing stream mesocosm channel unit and at the catchment scale. First we tested whether we could train ANNs using data from a stream mesocosm experiment that included varying nutrient and sediment levels. We then aimed to use these ANNs 1) to predict the nutrient and sediment status of river sites from their measured macroinvertebrate assemblages, and 2) to infer the relative significance of nutrient and sediment levels on the condition of river macroinvertebrate assemblages along a gradient of catchment area under grazing. These aims were achieved, demonstrating the value of using neural networks to distinguish landscape-scale impacts on rivers, and showing that pattern recognition can overcome the availability of only a relatively small amount of data.

## Methods

### Ethics Statement

This research did not involve vertebrates or cephalopods and therefore was not required to be approved by the Animal Ethics Committee of the University of Tasmania, which complies with the Australian Code of Practice for the Care and Use of Animals for Scientific Purposes (8^th^ Edition, 2013), the Tasmanian Animal Welfare Act 1993, and the Australian Code for the Responsible Conduct of Research (2007). All sites sampled (see S1 Table) were on private land, state forest or crown land and permits were not required. Forestry Tasmania who manage state forest and collaborated on this research confirmed that permits were not required for access to state forest and the Tasmanian Department of Primary Industries, Parks, Water and Environment who manage crown land confirmed that no permits were required for access to crown land. Private land owners gave permission for the study to be conducted on their land; land title details can be found on the Tasmanian Land Information System (‘The LIST’ https://www.thelist.tas.gov.au/app/content/home). Most taxa sampled in this research were identified to species and no taxa were listed as endangered or protected in Australian state or federal legislation.

### Selection of gradient survey sites

Potential stream sites in northern Tasmania, Australia, were screened by catchment size, northing and slope, and also according to attributes aimed at minimising confounding variables, maintaining broad consistency in landscape and geomorphological context, and promoting independence among sites (Table 1). A set of survey sites was selected across a gradient from low to high proportion of land under grazing in their upstream catchments. The extent of grazing land-use was determined from the Bureau of Rural Sciences land-use data layer held by the Department of Primary Industries, Parks, Water and Environment [35] and data on public production and plantation forests supplied by Forestry Tasmania. Land-use categories were based on amalgamations of the Australian Land-use and Management (ALUM) classification [36] (S2 Table). The total area (km^2^) of each land-use type in each catchment was calculated using ArcMap V.10 and converted to a percentage of the total catchment area.

**Table 1:** Selection criteria used to identify river sites in northern Tasmania suitable for sampling.

Selection criterion	Reasoning
Catchment size (20–120km2)	To minimise influence of ecosystem size
Lowland river (low riverbed slope)	To minimise influence of bedslope and channel morphology
Outside the Tasmanian World Heritage Area	To maximise influence of agricultural activity
No major water impoundments upstream	To minimise influence of confounding flow alteration, particularly due to power generation
Low proportion of granitic geology in the catchment (<30%)	To minimise influence of sandy, mobile substrata
Low proportion of mining and/or urbanisation (<5%) in catchment	To minimise influence of confounding stressors
Site catchments do not overlap each other	To maximise independence among sites
Site accessible	To enable benthic sampling
Presence of riffle habitat at the site	To enable relevance of mesocosm data

**Fig 1:**
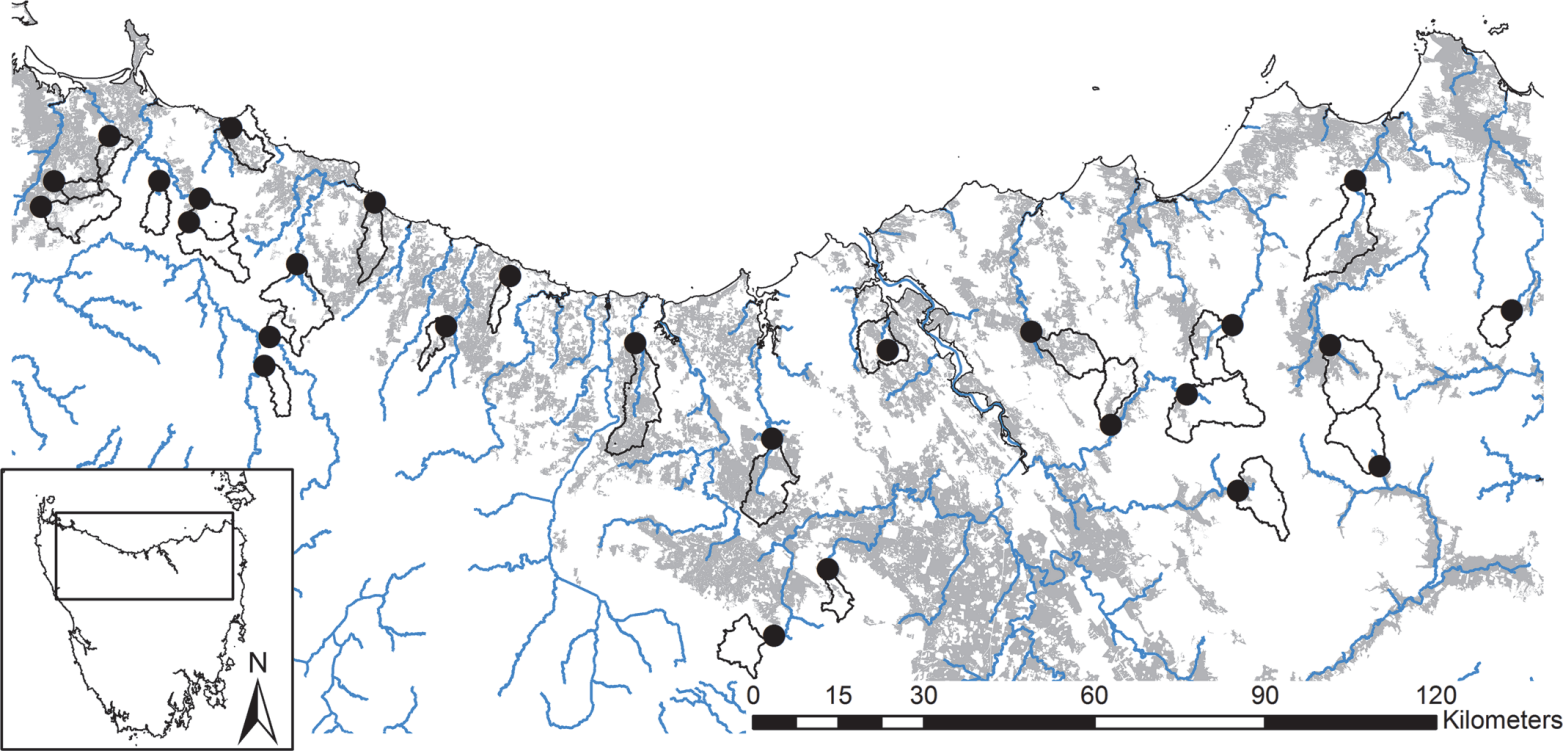
Map of study area and survey sites. Twenty-seven sites (●) were surveyed across northern Tasmania over two years. Site catchments were independent (black lines) and covered a gradient of grazing intensity (0 to 80% grazing of total catchment areas; grey shading shows land used for grazing livestock). Land-use data were supplied by DPIPWE (2009), and other spatial data were extracted from the Conservation of Freshwater Ecosystem Values database (DPIWE 2005) and the Land Information System Tasmania (theLIST) © Tasmania.

Twenty-seven sites were deemed suitable for the survey (S1 Table), with grazing representing 0 to 80% of the land-use in their upstream catchments; the areas of upstream catchments ranged from 20 to 120 km^2^ (stream order ranged from 4 to 6 [37]). Biological and physical data were sampled in riffle habitat at these 27 sites over the summers of 2008/2009 and 2009/2010 (Fig. 1). We did not detect any effect of year in any of our analyses, so data were pooled across years.

### Biological data

Macroinvertebrates were sampled using a 0.1 m^2^, 250 μm Surber sampler, and 15 samples were taken at random positions within the riffle zone at each site. Samples were fixed in 5% formalin before laboratory processing. All macroinvertebrates within a 20% sub-sample of the organic matter were identified to family and counted, with individuals from the insect orders Ephemeroptera, Plecoptera and Trichoptera identified to genus/species. Taxa were also categorised into functional feeding groups/traits (FFGs) according to information supplied by Environmental Protection Authority (EPA) Victoria (data sources: [38–41] and unpublished data from EPA Victoria). Community structure was analysed as a multivariate dataset by family and FFG. The conclusions from each approach were similar so only the FFG results are presented. Grazing land-use explained more variability in macroinvertebrate FFG traits than in taxonomy, and change in the functional structure of stream communities was deemed more likely to correlate with the processes driving the impacts of land-use [4, 42, 43]. We also analysed change in community structure using a commonly used univariate metric of macroinvertebrate community structure, the proportion of abundance represented by Ephemeroptera, Plecoptera and Trichoptera species (% EPT; [7, 44]).

Algal abundance was estimated at each site as the proportion of algal cover and as areal density of benthic chlorophyll *a*, and these were treated as predictor variables for macroinvertebrate community structure. Chlorophyll *a* was also later used as an input variable in the artificial neural networks. Proportion of algal cover was visually estimated to the nearest 0.25% from 15 replicate samples arbitrarily located within the riffle zone using a 0.1 m^2^ quadrat demarcated by wires into a 10 × 10 grid [45]. In addition, 15 arbitrarily located submerged cobbles in the riffle zone were selected for benthic chlorophyll *a* sampling. The upper surfaces of these cobbles were scoured using 45 mm^2^ surface area scourer pads [46], and the pads kept frozen and in the dark until processing. Chlorophyll *a* concentration (mg/m^2^) was estimated by pooling the 15 replicate samples and extracting pigments using 90% acetone; absorbance was measured at 663 nm for chlorophyll *a* and at 750 nm to remove the effect of turbidity [47].

### Environmental data

A set of physicochemical variables that had been identified in a separate data-mining exercise as potential land-use drivers of change in Tasmanian stream benthic macroinvertebrates [48] were sampled at each gradient survey site. Water temperature and conductivity were measured at the time of sampling, and under base flow conditions, with a WTW 315i conductivity meter and Tetracon 325 probe. Turbidity was measured with a Hach 2100P Turbidimeter, and water samples were collected for laboratory analyses of pH, total alkalinity, nitrate+nitrite (NO_x_), dissolved reactive phosphorus (DRP), total nitrogen (TN) and total phosphorus (TP; Analytical Services Tasmania). Overhead shading was measured with a hemispherical densiometer as proportional canopy cover, and the proportion of fine sediments within the sampled riffle zone was estimated using the AusRivAS rapid assessment protocol for substratum composition (percent cover of sand (0.06–2 mm) and silt (< 0.06 mm) [49, 50]). Two variables representing flow modification were sourced from Tasmania’s Conservation of Freshwater Ecosystem Values (CFEV) database [51], in turn derived from data held in the Water Information System of Tasmania (WIST) and by Hydro Tasmania. The “accumulated abstraction index” was the sum of all upstream abstractions and diversions divided by the long-term (20–35 year) mean annual runoff, and the “accumulated regulation index” was the sum of all upstream catchment storages (e.g. farm dams) divided by the long-term mean annual runoff.

### Survey analysis: linking biodiversity data with environmental data

A distance-based redundancy analysis (dbRDA) [52] was used to identify the environmental variables that explained the greatest variation in macroinvertebrate FFG (community structure) data at the gradient sites. Between-site Bray-Curtis similarities [53] index values were calculated from a matrix of macroinvertebrate taxon by abundance data, with abundance data square-root transformed to reduce the influence of numerically abundant species. The environmental data were normalised. Analyses were conducted in PRIMER V6.1.12 with the PERMANOVA+ V1.0.3 add-on [54].

Several of the predictor variables were highly inter-correlated: alkalinity and conductivity (*r* = 0.96), regulation and abstraction (*r* = 0.92), and TP and DRP (*r* = 0.96), so we excluded the variable in each pair that was least correlated with the dbRDA axes, and hereafter refer to Conductivity/Alkalinity, Abstraction/Regulation and DRP/TP respectively. This resulted in 10 environmental variables being used to explain variation in macroinvertebrate communities and algal biomass across a gradient of grazing land-use.

### Stream mesocosm experiment

Two stream mesocosm experiments were conducted in the summer-autumn of 1996 and 1997 to distinguish the influence of fine sediment loads and nutrient concentrations on benthic macroinvertebrate and algal communities (P.E. Davies and L.S.Cook, unpubl. data). The experiments were conducted using a flow-through water supply via diversion and hydraulic manifold from the Little Denison River, Tasmania (-43.0, 146.8), which was similar in geomorphology and hydrology to the gradient survey sites. In each experiment, 32 flow-through mesocosms, each 4 m length × 0.4 m width × 0.4 m depth, were established with cleaned cobbles sourced from the adjacent river and colonised for four months by continuous constant flow-through from the Little Denison River.

In a split-plot design, the mesocosms received low or high nutrient concentrations (Low: 0.035 mg/L NO_3_-N and 0.008 mg/L PO_4_-P; High: 0.4 mg/L NO_3_-N and 0.08 mg/L PO_4_-P), and low or high fine sediment loads (Low: < 5 g/m^2^ sand (grain size 0.06–2 mm) initially and fortnightly pulses of < 5 mg/L suspended clay (grain size <0.06 mm) during 12 hr of raised flows; High: 1 kg/m^2^ sand initially and fortnightly pulses of 100 mg/L suspended clay). These concentrations were selected based on field measurements of streams impacted by agriculture and forestry ([55]; P.E. Davies, unpubl. data). Nutrient conditions were not sufficiently low to be limiting in the control treatments, and in the high nutrient treatments were representative of high mean values in Tasmanian agricultural catchments (baseflow values: TN: 0.01–1.5 mg/L; TP: 0.01–0.05 mg/L [56]). Benthic sediment loads in the high sediment treatments were similar to those observed in Tasmanian streams in highly grazed and cleared agricultural catchments. There were 16 replicates of each treatment combination, and each experiment ran for 90 days. One macroinvertebrate and one composite benthic algal sample was collected from each mesocosm at the end of each experiment by Surber and scrape sampling as detailed above, resulting in 64 samples in total. Benthic algal biomass (chlorophyll *a*) and abundance of most of the macroinvertebrates used in this study were not significantly different between the two years so the two data sets were combined (1-way ANOVAs conducted on control treatments all *P* >0.1 except for *Nousia* spp. (*F*_1,6_ = 17.5, *P* = 0.006) and *Austrophleboides* spp. (*F*_1,6_ = 14.1, *P* = 0.01), P.E. Davies, unpubl. data). A third factor, ‘Light’, was also manipulated in the mesocosm experiment with the use of commercially available shade-cloth over half the replicate stream channels. This factor was ignored for the purpose of training ANNs because it was difficult to equate the use of shade cloth with local and catchment scale riparian shading in the gradient survey. To reiterate, our purpose was not to fully analyse the results of the mesocosm experiment, but to use the mesocosm data to train the ANNs. Therefore, the ANNs were trained to find patterns in macroinvertebrate community composition correlated with sediment and nutrient concentrations irrespective of year and light exposure.

Eleven variables were considered to adequately describe the biological response to changes in the nutrient and sediment levels: chlorophyll *a* (mg/m^2^), macroinvertebrate familial richness, total abundance, and the abundance of Oligochaeta, *Leptoperla varia* (Gripopterygidae), *Nousia* spp. (Leptophlebiidae), *Austrophlebioides* spp. (Leptophlebiidae), Orthocladiinae, Tanypodinae, Tipulidae and larval Scirtidae. These taxa were abundant within and among the stream mesocosm communities and are common in a wide range of Tasmanian rivers including streams in the gradient survey study area. Values for each of 11 biological response variables were standardised by dividing by their average value observed in the experimental controls mesocosm samples from that year. Experimental treatment effects were examined using 2-way ANOVAs in R prior to training the ANNs [57], with the aim of determining whether a standard statistical approach could detect effects within the dataset rather than developing a complete statistical model.

### Simplifying the pattern recognition exercise

The stream mesocosm experiments together produced 64 records. Nutrient and sediment levels were assigned values of 1 (low levels) and 2 (high levels) respectively. The standardised values of the 11 biological response variables were then rounded to the nearest integer, ranging from -2 to +3.

In the pattern recognition exercise, the 11 biological response variables were the inputs to the ANNs, and the nutrient and sediment levels were the outputs (the opposite of the presumed causal relationships). The preferred strategy if enough training and validation data were available would be to produce ANNs linking all 11 inputs to both outputs. However, with only 64 records available it was necessary to simplify the pattern recognition exercise. We found that separate ANNs to predict nutrient and sediment levels performed quite well, especially if two of the 11 biological response variable inputs were discarded on the basis of low correlation with the target variable. For the nutrient level ANNs, the abundance of *Nousia* spp. and the abundance of Tanypodinae were discarded. For the sediment level ANNs, total abundance and the abundance of *Austrophlebioides* were discarded. We used 32 records to train each ANN, and used the remaining 32 records to evaluate the ANN’s performance. The 32 records were chosen to span the -2 to +3 input data ranges to the extent possible, with priority given to inputs with highest correlations to the target output variable. For the sediment level ANNs, the priority inputs were the abundance of *Nousia* spp., macroinvertebrate familial richness, and the abundance of Orthocladiinae. For the nutrient level ANNs, the priority inputs were chlorophyll *a*, the abundance of larval Scirtidae, and macroinvertebrate familial richness.

### Artificial neural network architecture and training

A three layer feed-forward ANN was used (see [58, 59] for an introduction to artificial neural network theory). In brief, a three layer ANN has an input layer of neurons, a hidden layer of neurons, and a single output layer neuron. Neurons in the input layer accept a set of inputs (*x*_*i*_), apply a weight (*w*_*i*_) to each input, sum the weighted inputs, add a bias (*b*), and the neuron’s transfer function, *f*, then produces an output *y = f* (Σ*w*_*i*_ *x*_*i*_ *+ b*). Similarly, each neuron in the hidden layer accepts the outputs of all the input layer neurons, and produces its own output, which is directed to the output layer neuron. Tangent-sigmoidal or log-sigmoidal transfer functions are often chosen for input and hidden layer neurons for applications such as this, where the exact form of the transfer function is not important, beyond needing to have a continuous output (i.e. not be a step-function). The weakly non-linear S-shaped parts of these transfer functions lie between input values of about -3 to +3 (tan-sig) and about -5 to +3 (log-sig), and produce an output between -1 and +1 (tan-sig) or 0 and 1 (log-sig). Lower or higher input values produce constant responses of -1 and +1 respectively. We chose tangent-sigmoidal transfer functions for both the input and hidden layer neurons.

There are several ways in which an ANN can produce an output which can only have one of two values. Using a single output neuron with a step-function transfer function is one option, but we decided to use a single output neuron with a linear transfer function. The neuron accepts inputs from all the hidden layer neurons, and applies the linear transfer function to produce the ANN’s output. We trained the ANN to produce an output of 1 or 2 as appropriate, but the use of a linear transfer function means the output value is not constrained to be either 1 or 2, and its proximity to one or other of these values is a measure of the confidence of the ANN in that prediction.

Each ANN was trained in batch learning mode using the Matlab back-propagation algorithm *trainlm*, which compares the ANN's predictions for all the training data records to the required output values (1 or 2), and adjusts the neuron weights and biases using gradient descent, momentum and an adaptive learning rate, working backwards from the output layer to the input layer [60]. The ANN training was stopped when the mean squared error (MSE) of the residuals reduced to 0.2, which usually took about 100 training epochs (an epoch refers to the cycle of making predictions, examining the residuals, and adjusting the neuron weights and biases, and the training algorithm parameters). A smaller MSE training goal would risk producing an ANN that overfits the training data set, and is not able to generalise.

Both the nutrient level and sediment level ANNs use 6 input layer neurons and 3 hidden layer neurons, which experimentation found to be the simplest architecture able to identify the pattern in the input data (to help avoid overfitting, it is good practice not to use more layers or neurons than is necessary).

A final component of the strategy to deal with the problem of having a small training data set was to produce 50 ANNs to predict the nutrient level, and another 50 ANNs to predict the sediment level. The initial weights and biases in the ANNs are partly random in nature, so two training exercises will not proceed to alter them in quite the same way. With 9 inputs and only 32 training records, it is expected that different ANNs can achieve the MSE training goal by focusing on different input variables. Training 50 ANNs and then applying all of them to validation or new data results in better pattern recognition performance than is likely to be achieved by any single ANN. The result of the pattern recognition exercise was the mean predicted nutrient or sediment level (+/- standard deviation).

### Diagnosis of grazing impacts in gradient survey using ANNs

**Fig 2:**
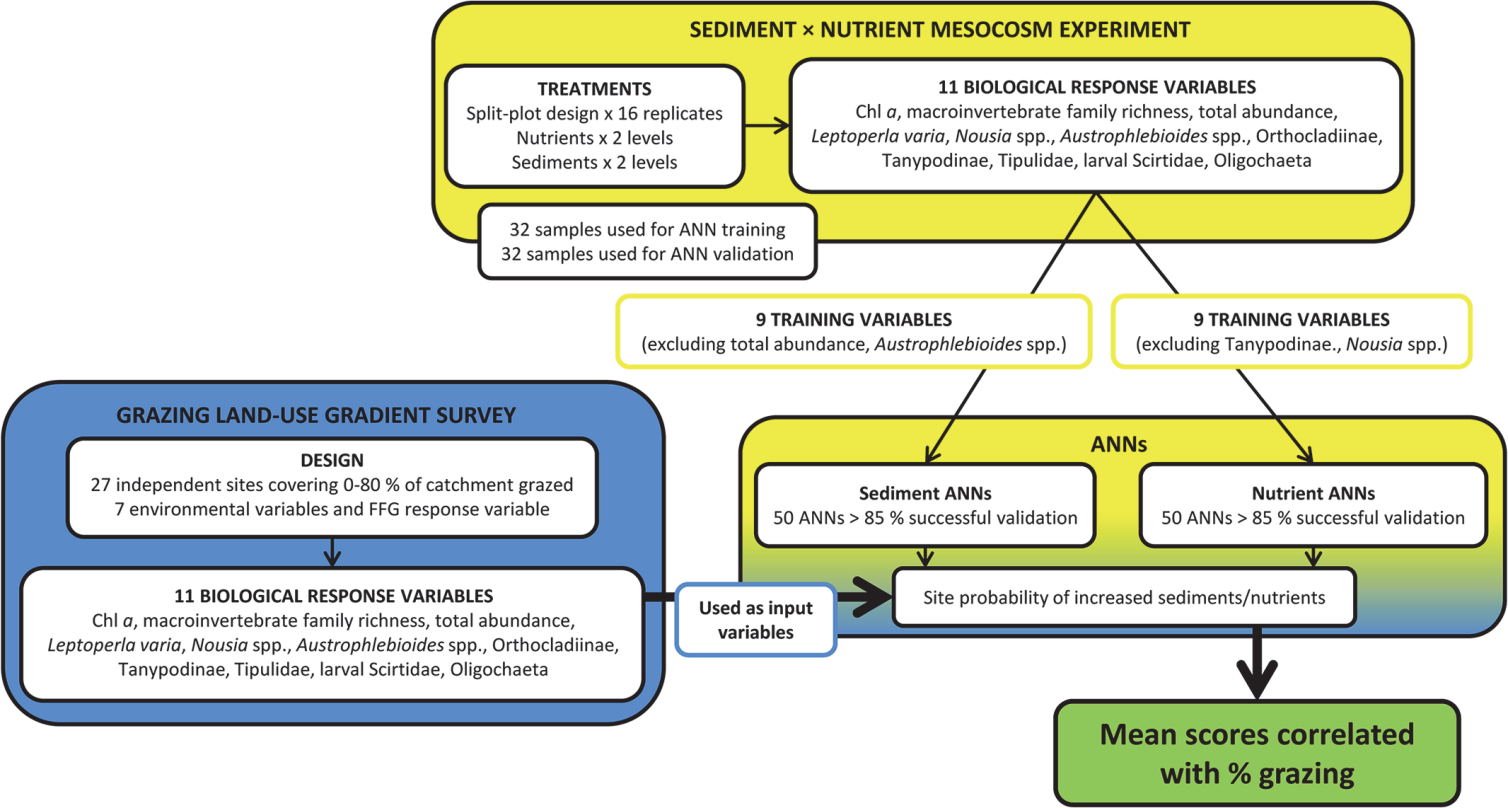
Data flow between gradient survey, mesocosm experiment and artificial neural networks (ANNs). Sites were sampled across a gradient of grazing intensity, expressed as the area proportion of the catchment used for grazing livestock. The sediment and nutrient ANNs were constructed and trained on response variables from a mesocosm experiment run at varying sediment and nutrient levels, with half these data held back for ANN validation. The validated ANNs were then used to generate probabilities of site sediment and nutrient status based on the site biological response variables measured in the gradient survey.

The values of the biological input variables used for ANN training (9 variables for each of the sediment and nutrient ANNs) were extracted from the gradient survey data for each survey site, and standardised by dividing by the average value measured among ‘control’ sites with <1% grazing in their catchments (Fig. 2). These field-derived variables were then entered into each of the ANNs to generate a probability of each site experiencing increased fine sediment loads and nutrient concentrations (Fig. 2). The probabilities were used to derive a linearly-interpolated score from 1 (low predicted fine sediments or nutrients) to 2 (high predicted fine sediments or nutrients). Average probabilities were computed for each site from the 50 ANN predictions, and correlated with the area proportion of grazing land in the catchment upstream of each site using linear regression in R [57].

A poor correlation between the ANN output scores and the proportion of catchment grazing land-use would imply that fine sediments or nutrients were either not correlated with grazing and/or that the ANNs did not adequately represent the ‘real world’ response of river biota to fine sediment and nutrient increases. Alternatively, a high correlation between ANN output scores and the proportion of catchment grazing land-use would indicate that fine sediments or nutrients were likely to be causal in describing biological response to grazing in the catchment *and* that ANNs are useful for examining ecological responses to fine sediments or nutrients in rivers at the landscape scale.

## Results

### Biological response to grazing (gradient survey)

**Fig 3:**
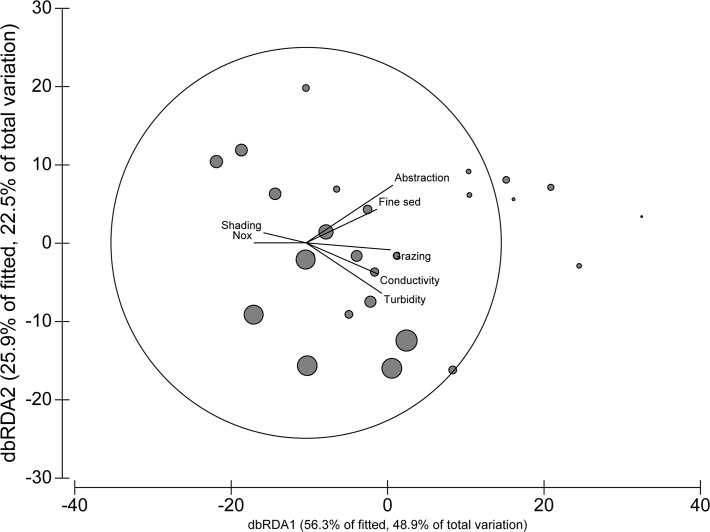
Ordination plot of sites separated by relative abundance of functional feeding groups. The distance-based redundancy analysis was constrained by the environmental data. The lengths of the overlaying vectors are proportional to the multiple partial correlations of each environmental variable with dbRDA1 (the circle represents the maximum vector length, a correlation of |1|) and the direction of the vectors illustrates both the direction of the correlation with dbRDA1 and the degree of correlation with dbRDA2. Only correlations >|0.2| with dbRDA1 are displayed. Symbol size represents the abundance of invertebrates classified as ‘leaf shredders’.

**Fig 4:**
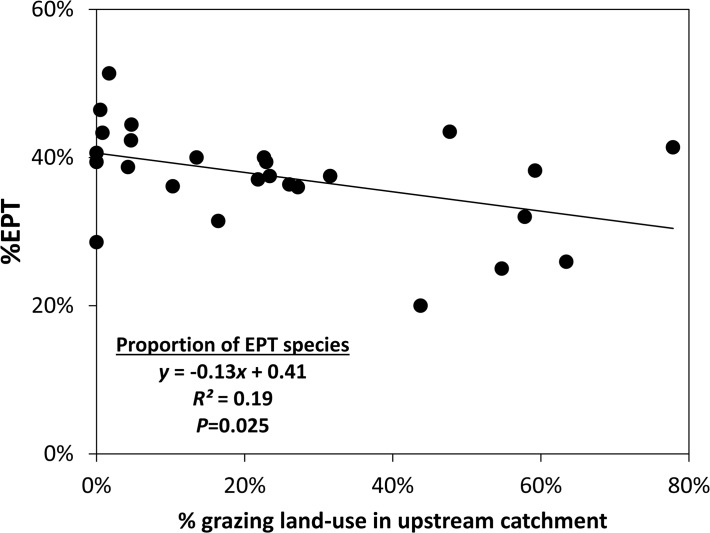
Relationship between %EPT and proportion of upstream catchment subject to grazing.

The area proportion of grazing land-use in catchments upstream of survey sites varied from 0 to 80%, and was well correlated with macroinvertebrate community structure as defined by functional feeding groups (FFG; Fig. 3) and percent EPT (Fig. 4). The multiple partial correlation (mpc) between grazing land-use and the first dbRDA axis was 0.44 (Fig. 3). The abundance of invertebrates classified as leaf shredders (1–285 individuals) was used to illustrate the change in FFGs: the change in abundance of leaf shredders was negatively correlated with the first dbRDA axis and therefore with the area proportion of grazing land-use (Fig. 3). Percent EPT varied from 20 to 51% and was significantly negatively correlated with the area proportion of grazing land-use (Fig. 4).

The environmental variables that were well correlated with macroinvertebrate community structure and the area proportion of grazing land-use included abstraction/regulation (0 to 4% and 0 to 7% of mean annual runoff respectively; mpc = 0.45), turbidity (0 to 10.5 NTU; mpc = 0.39), and alkalinity/conductivity (0 to 196 mg-CaCO_3_/L and 42 to 481 μS/cm respectively; mpc = 0.37) (Fig. 3). The proportion of substrate area represented by fine sediments varied from 0 to 45% and was well correlated with community structure (mpc = 0.36), while the three nutrient variables (NO_x_ (0 to 0.82 mg/L), DRP (0 to 0.041 mg/L) and TN (0 to 1.3 mg/L)) were weakly correlated with the first dbRDA axis (all |mpc| <0.3), with NO_x_ actually showing a negative relationship with the first dbRDA axis and therefore with the area proportion of grazing land-use (Fig. 3).

### Responses to mesocosm experimental treatments

The stream mesocosm experiments were conducted to distinguish the influence of fine sediment loads and nutrient concentrations on benthic macroinvertebrate communities and algal coverage. The 11 biological response variables displayed varying degrees of sensitivity to elevated levels of fine sediments and/or nutrients, although the majority were not statistically significant (9 variables all *F*_1,60_ < 3.11, *P* > 0.08). Only two significant treatment effects were detected: chlorophyll *a* was significantly higher with increased concentration of nutrients only (*F*_1,60_ = 8.2, *P* = 0.006), and the standardised abundance of Orthocladiinae was significantly higher with increased fine sediments only (*F*_1,60_ = 9.87, *P* = 0.003). We did not detect a significant interaction between sediments and nutrients for any of the 11 biological response variables examined (all *F*_1,60_ < 1.55, *P* > 0.22).

### Neural network results

**Fig 5:**
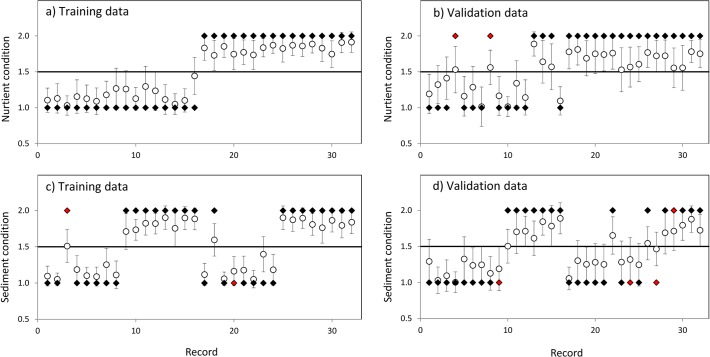
Validation results for ANNs trained to predict nutrient or fine sediment condition. Predictions from nutrient ANNs were generated from training (a) and validation (b) datasets. Predictions from sediment ANNs were generated from training (c) and validation (d) datasets. Each pair of points represents one mesocosm (labelled ‘Record’): the white circle is plotted at the mean (+/- standard deviation) of the condition scores predicted by 50 ANNs, while the black diamond plots the actual condition (nutrient or sediment level) of each mesocosm as 1 (low) or 2 (high). Diamonds coloured red indicate an incorrect prediction.

The ANNs trained on stream mesocosm training data sets performed well in predicting sediment and nutrient status in the stream mesocosm validation data sets. The mean predictions of the 50 nutrient ANNs correctly predicted nutrient status for all the training data set records, and for 30 of the 32 observations in the validation data set (error rate = 6.3%; Fig. 5). The mean predictions of the 50 sediment ANNs correctly identified sediment status for all but two training data set records, and for 28 of the 32 observations in the validation data set (error rate = 12.5%; Fig. 5).

The trained and validated ANNs were then used to predict the nutrient and sediment status of the gradient survey sites from the values of the 11 biological response variables recorded at these sites.

**Fig 6:**
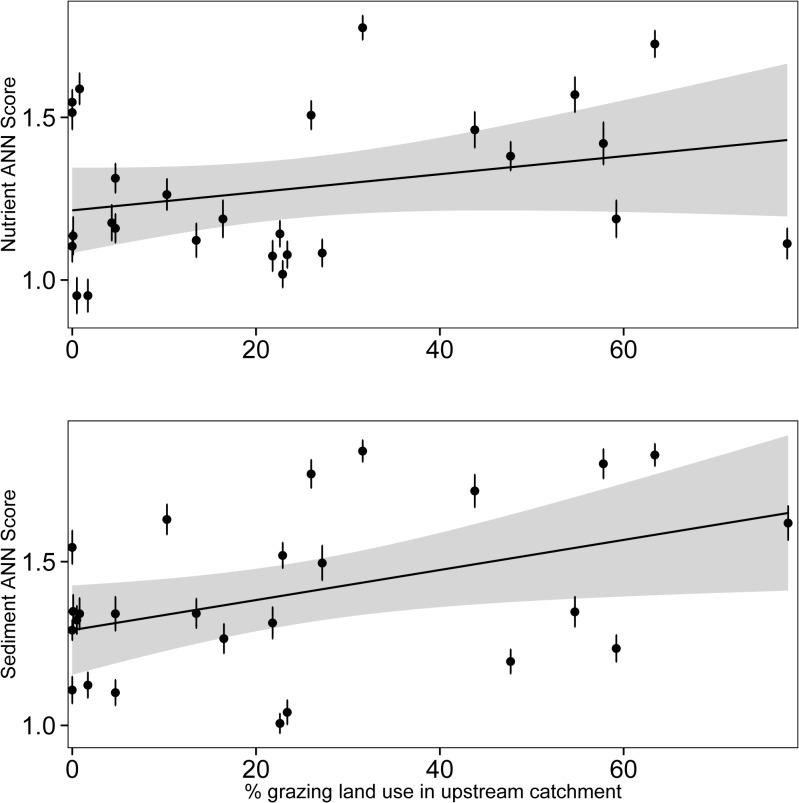
Relationships between ANN predictions and the area proportion of grazing in the catchment. Linear regressions are of predicted scores from nutrient (a) and sediment (b) ANNs against the area proportion of grazing in the catchment. Points plotted are mean (± standard error) ANN output scores. Grey shading represents standard error of the linear fit. Linear fits show a significant relationship for fine sediments (*R* ^2^ = 0.18, *P* < 0.03) but not for nutrients (*R* ^2^ = 0.08, *P* = 0.164).

The predicted nutrient status of the gradient survey sites was not significantly related to the proportion of grazing land-use in the upstream catchments (*F*_1,26_ = 2.06, *P* = 0.164; Fig. 6). In contrast, the predicted sediment status of the gradient survey sites was significantly related to the area proportion of grazing land-use in the upstream catchments (*F*_1,26_ = 5.42, *P* < 0.03), although the goodness of fit was poor (*R* ^2^ = 0.18; Fig. 6).

## Discussion

Our study shows that ANNs trained on independent experimental data sets can be a useful diagnostic tool for disentangling the effects of often-confounded variables in a landscape-level data set. Using ANNs, we were able to detect subtle influences undetectable using common tests for significant correlation, and identify fine sediments as a stronger mechanism than nutrients for mediating grazing land-use impacts on macroinvertebrate communities. Further, we demonstrated that ANNs can be trained on relatively small and noisy datasets, providing a means of extrapolating small-scale experimental findings to explain landscape-scale patterns. Given the popularity of mesocosm-scale experiments during the 1990s [20], our approach demonstrates the potential for applying these existing datasets to diagnose landscape patterns and impacts, provided the general biophysical contexts of the experiment and the analysed landscape are sufficiently similar.

In our field survey, univariate and multivariate indices of macroinvertebrate community structure were well correlated with the proportion of grazing land-use and with several of the physical variables closely associated with grazing impacts; abstraction and regulation, turbidity, conductivity and alkalinity, and the proportion of fine sediments in the substratum. Our results are consistent with the large number of studies examining impacts of agricultural land-use on stream macroinvertebrates [48, 61, 62]. Our subsequent key finding was that ANNs allowed us to distinguish the relative influence on macroinvertebrate assemblages of sediment and nutrient inputs associated with agricultural land-use impacts. Sediment and nutrient inputs are rarely adequately measured in routine or snapshot monitoring and are often confounded with each other and other landscape variables, leading to difficulties in identifying mechanisms of land-use impacts without applying large-scale costly experiments [63, 64]. It is difficult to adequately describe fine sediment impacts in a snapshot survey because the potential causal pathways for impact of fine sediment on stream communities vary [65] and because the relationship between flow and sediment transport is characterized by hysteresis [66]. Furthermore, even in experimental settings macroinvertebrate assemblages can respond to both sediments and nutrients, making it difficult to determine the relative importance of either [64, 67]. By training ANNs on small-scale experimental data, we have added evidentiary support to the identification of drivers of biotic responses observed by standard statistical analyses of our gradient survey data. We have also developed a tool, likely constrained to local application, for reliably and independently predicting sediment and nutrient status in the field from macroinvertebrate community data. This demonstrates that the cause-effect relationships between the land-use variables and the physicochemical and biological variables used to train our ANNs were independent of spatial scale, allowing its application across multiple spatial scales.

Our finding that sediments have a detectable influence on macroinvertebrate assemblages supports previous studies [64, 67], but our results are inconclusive for nutrient impacts. This could have occurred due to 1) a lack of statistical power in the gradient survey because spot nutrient data was highly variable, 2) the nutrient ANNs having no generality at the landscape-scale, and/or 3) the biological indices selected in our gradient survey and mesocosm experiment being insensitive to changes in nutrient concentration. It is also possible that nutrient loads in northern Tasmanian rivers are not sufficient to impact river condition or that it is just too difficult to detect the impact of nutrients because the factors influencing nutrient concentrations are so complex. Bende-Michl *et al*. [68] showed that monthly water samples could not be used to describe nutrient dynamics in the Duck River (Tasmania, Australia) because much of the nitrogen and phosphorus transport was driven by rainfall events; both the magnitude of the individual rainfall event and the recent history of rainfall events influenced nutrient concentration. Alternatively, the importance of nutrients in driving stream condition may be masked by interactions with other factors (e.g. light availability). Clapcott et al. 2011 [62] were able to describe a threshold-type response of the median annual concentration of nitrate+nitrite to vegetation removal in a large-scale land-use gradient survey in New Zealand, but only after more than 80% of vegetation was removed from the catchment, and in that work there was no clear response to nitrogen in any of the macroinvertebrate response variables examined. Despite numerous survey and experimental studies, it appears that even using a subtle pattern-detection tool like an ANN, it is still difficult to clearly discern the direct effects of nutrients on stream condition.

On the other hand, the fact that we were able to show effects of sediment inputs suggests that sediments may be a stronger (and thus more detectable) mechanism mediating land-use impacts on macroinvertebrate communities. However, while we were able to show a significant impact of fine sediments across the grazing land-use gradient with the use of ANNs, the goodness of fit between the predicted degree of fine sediment impact and the proportion of upstream grazing land-use was poor. There are several possible sources of variance in this relationship. First, the amount of data generated in the mesocosm experiments could have been insufficient to train highly accurate ANNs, although our ANNs were successful in diagnosing condition status in the field. Second, the mismatch in timing between the mesocosm experiment (1996–1997) and the gradient survey (2008–2010) may have influenced the ecological response to fine sediments; and third, the artificial streams used in the experiments may not have been representative of the streams sampled in the gradient survey. If any of these points are responsible for some of the variance, then it will be possible to develop better diagnostic tools with more careful experimental design of both mesocosm and gradient surveys. The poor goodness of fit may also be due to natural variability in the biological data (mesocosm and/or gradient survey) and/or variability in the impact of grazing. If so, then the approach may only be useful when the impact is large.

Nevertheless, we have confidence in these findings because the field survey explicitly restricted sampling to sites with independent (non-overlapping) upstream catchments. This is rarely the case in studies exploring land-use impacts on rivers, yet field surveys without this restriction are essentially pseudoreplicated [69]. We have shown that, with careful site selection to avoid additional sources of variability and with the use of a diagnostic tool to interpret ecological impact, it should not be necessary to compromise site independence: a selection of 27 sites across an environmental gradient had sufficient statistical power to describe a response to land-use and identify potential causal drivers.

We were fortunate to have relatively simple relationships between our biological and physical variables. Complex responses such as synergistic effects and subsidy-stress responses [70] have been shown in aquatic communities using similar physical variables [6, 43, 67], but we found no evidence of such complex relationships perhaps because the patterns we observed were subtle. If complex relationships did exist they did not impair our ability to detect the effects of fine sediments. Diagnosing complex relationships using ANNs would require larger training datasets and probably more neurons in the input and hidden layers of the ANNs; in addition, more field sites would be required if the aim were to describe the nature of a complex relationship rather than simply to detect an impact.

We have shown that it is possible to successfully train ANNs on small datasets by carefully training a large number of replicate networks [50] and interpreting the average response, by training separate ANNs for each output variable (nutrients and sediments), an approach which performs better than training one ANN to predicts all the variables by excluding input (biological) data that was not well correlated with the output variables. As with any modelling tool, careful selection of the input data is important and will depend on the objectives of the research [71]. For example, good biological indicators of environmental impact that are static in space and time but sensitive to impact [72, 73] are likely to be useful in training scale-independent ANNs, but this may not be the case if the indicator was designed for a broader purpose [74]. The main caveat when applying an ANN to ecological situations is that its performance is necessarily dependent on the extent to which the training data capture the pattern being sought.

While we used ANNs to diagnose land-use impacts on stream macroinvertebrate communities, our approach of training networks that are independent of the spatial scale of observation could be used to interpret patterns in any ecological data. Neural networks could, therefore, be trained on multiple small experimental datasets, with appropriate indicators, to extrapolate patterns to larger spatial scales, but also to detect a range of ecological impacts. Incorporating ANNs into regular ecosystem monitoring could improve our ability to detect impacts in field survey data without increasing costs.

## Supporting Information

S1 Table List of 27 sites surveyed in the gradient study. (DOCX)

S2 Table List of land-use categories and abbreviations included in statistical analyses. Descriptions include references to the equivalent Australian Land-use and Management categories (ALUM; BRS 2006).
(DOCX)
