# Supplementary material for: Inferring Landscape-Scale Land-Use Impacts on Rivers Using Data from Mesocosm Experiments and Artificial Neural Networks
Source: PLoS One. 2015 Mar 16;10(3):e0120901. doi: 10.1371/journal.pone.0120901 (PMC4361191; doi:10.1371/journal.pone.0120901)
Supplement: S1 Table — (DOCX) [file pone.0120901.s001.docx]

# Supporting information file

Regina H. Magierowski, Steve M. Read, Steven J.B. Carter, Danielle M. Warfe, Laurie S. Cook, Edward C. Lefroy and Peter E. Davies.

**S1 Table.**  **List of 27 sites surveyed in the gradient study.**

| **Site** | **Stream Order** | **Easting** | **Northing** |
| --- | --- | --- | --- |
| Ansons river | 5 | 595978 | 5440636 |
| Black river | 5 | 358304 | 5463355 |
| Blackwell creek | 4 | 377714 | 5436002 |
| Boobyalla river | 5 | 568467 | 5463417 |
| Dans rivulet | 5 | 572699 | 5413337 |
| Deep creek | 4 | 349512 | 5471224 |
| Dip river | 4 | 363564 | 5456094 |
| Don river | 5 | 441960 | 5434904 |
| Dorset river | 5 | 564017 | 5434532 |
| Duck river | 4 | 337490 | 5458815 |
| Edith creek | 4 | 339837 | 5463277 |
| Ford river | 5 | 547855 | 5409005 |
| Gibson creek | 5 | 408680 | 5437904 |
| Great Forester river | 5 | 546874 | 5438028 |
| Inglis river | 6 | 382523 | 5448770 |
| Meander river | 4 | 466337 | 5383531 |
| Melin rivulet | 5 | 365434 | 5460248 |
| Parrawe creek | 4 | 376761 | 5430942 |
| Patersonia rivulet | 4 | 525470 | 5420564 |
| Penguin creek | 4 | 419987 | 5446658 |
| Quamby Brook | 4 | 475798 | 5395204 |
| Rubicon river | 5 | 465958 | 5418184 |
| Salisbury Creek | 5 | 486444 | 543890 |
| Seabrook creek | 4 | 396163 | 5459547 |
| Second river | 4 | 511529 | 5436905 |
| St Patricks River | 6 | 538902 | 5425908 |
| Wilsons creek | 4 | 370975 | 5472610 |
