# Supplementary material for: Inferring Landscape-Scale Land-Use Impacts on Rivers Using Data from Mesocosm Experiments and Artificial Neural Networks
Source: PLoS One. 2015 Mar 16;10(3):e0120901. doi: 10.1371/journal.pone.0120901 (PMC4361191; doi:10.1371/journal.pone.0120901)
Supplement: S2 Table — Descriptions include references to the equivalent Australian Land-use and Management categories (ALUM; BRS 2006). (DOCX) [file pone.0120901.s002.docx]

# Supporting information file

Regina H. Magierowski, Steve M. Read, Steven J.B. Carter, Danielle M. Warfe, Laurie S. Cook, Edward C. Lefroy and Peter E. Davies.

**S2 Table.** **List of land-use categories and abbreviations included in statistical analyses.** Descriptions include references to the equivalent Australian Land-use and Management categories (ALUM; BRS 2006).

| Land-use category | Abbreviation | Description |
| --- | --- | --- |
| Non-production native vegetation | Minimal-use | Nature conservation, managed resource protection and other minimal use. Includes all formal and informal reserves, and any areas of intact native vegetation. It also includes all aspects of non-production forest, such as formal and informal reserves on private and public land.  ALUM codes 1.1, 1.2 and 1.3. |
| Grazing by domestic live stock | Grazing | Grazing natural vegetation and grazing modified pastures. Represents all forms of extensive and moderate intensity grazing land management including dairy farming.  ALUM 2.1 and 3.2 and 5.2.1 |
| Production forestry | Production | Commercial production from native forests and related activities on public and private land.  ALUM code 2.2. |
| Plantation forestry | Plantation | Land on which plantations of trees or shrubs (native or exotic species) have been established for production or environmental and resource protection purposes.  ALUM code 3.1. |
| Cropping and horticulture | Cropping | Includes cropping, irrigated perennial and seasonal horticulture and intensive plant production e.g. glasshouses.  ALUM codes 3.3, 4.3, 4.4, 4.5 and 5.1 |
